# KSQ‐4279, an Inhibitor of Ubiquitin Specific Peptidase 1, Enhanced the Chemotherapeutic Efficacy in ABCB1/ABCG2/ABCC1‐Mediated Multidrug Resistant Cancers

**DOI:** 10.1002/mco2.70517

**Published:** 2025-11-29

**Authors:** Qihong Yang, Kewang Luo, Kenneth Kin Wah To, Can Pan, Kai Fu, Shuangli Zhu, Sijia Li, Fang Wang, Chuanan Wu, Liwu Fu

**Affiliations:** ^1^ State Key Laboratory of Oncology in South China Guangdong Provincial Clinical Research Center For Cancer Sun Yat‐sen University Cancer Center Guangzhou P. R. China; ^2^ People's Hospital of Longhua Shenzhen P. R. China; ^3^ School of Pharmacy The Chinese University of Hong Kong Hong Kong P. R. China

**Keywords:** ATP‐binding cassette transporters, combination chemotherapy, multidrug resistance, KSQ‐4279, ubiquitin specific peptidase 1

## Abstract

Multidrug resistance (MDR) remarkably hinders the success of clinical chemotherapy in carcinomas. The ATP‐binding cassette (ABC) transporter as ABCB1, ABCG2, and ABCC1 are most crucial to drive MDR. Regrettably, no MDR modulators have been accepted in clinic. Herein, KSQ‐4279, a first‐in‐class ubiquitin‐specific peptidase 1 (USP1) inhibitor in clinical development, was found as a pan‐MDR modulator. Our study showed that KSQ‐4279 strikingly intensified the cytotoxicity of multiple classical chemotherapeutic drugs in ABCB1/ABCG2/ABCC1‐induced MDR cancers independent of its own cytotoxicity in vitro, and remarkably improved chemotherapeutic efficacy not only in ABCB1/ABCG2/ABCC1‐overexpressing tumor xenografts in vivo, but also in ABCB1‐overexpressing clinical lung cancers ex vivo. Mechanistically, KSQ‐4279 weakened ABCB1/ABCG2/ABCC1 efflux function, thus increasing drugs’ reservation in cells; more specifically, it was achieved by KSQ‐4279 activating the ATPase activity and competing for the substrate‐binding pockets of ABCB1/ABCG2/ABCC1. Besides, at the effective reversal concentrations, KSQ‐4279 neither altered expression and localization of ABCB1/ABCG2/ABCC1, nor affected USP1's potential downstream AKT or ERK1/2 signaling. This is the first study to investigate the combination of USP1 inhibitor (KSQ‐4279) with traditional chemotherapeutic drugs in reversing MDR, which surprisingly hinted ABCB1, ABCG2, and ABCC1 as the new targets of KSQ‐4279, and advocated this promising combination therapy in clinical refractory MDR cancers.

## Graphical abstract

KSQ‐4279, an USP1 inhibitor in clinical trial, widely reversed multidrug resistance in refractory cancers by competing for the drug‐binding pockets of ABCB1/ABCG2/ABCC1 and blocking chemotherapeutic drugs’ efflux.

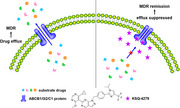

## 1 Introduction

Chemotherapy is the most common treatment modality for cancer therapy. Despite the discovery of numerous novel chemotherapeutic drugs, clinical studies showed that multidrug resistance (MDR) is severely hindering their efficacy in cancer patients [1]. Indeed, in cancer patients receiving conventional chemotherapy or targeted therapies, the majority of cancer‐related mortalities (exceeding 90%) are attributed to MDR [2]. To date, there is no safe and effective clinically approved MDR modulator to handle the treatment failure cases.

The ATP‐binding cassette (ABC) drug efflux transporters are mainly located at cell membrane and their overexpression is widely recognized as the most common and critical mechanism driving MDR [3]. Highly conserved in structure, ABC transporters are usually made up of four domains, including two cytosolic domains to couple nucleotides (NBDs) and two transmembrane domains to bind substrates (TMDs) [4]. When the TMDs bind with the substrates (chemotherapeutic drugs), the conformation of ABC transporters would be changed, thereby facilitating the hydrolysis of ATPs by the NBDs to provide energy for transporting substrates out [5]. In human, 49 ABC transporter members have been authenticated and further categorized into seven families according to their genetic sequences and substrate selectivity [6]. Among ABC transporters triggering MDR, subfamilies of B1 (ABCB1), G2 (ABCG2), and C1 (ABCC1) have been widely investigated in‐depth. ABCB1 was reported to promote MDR to plenty of conventional anticancer agents, including vincristine, doxorubicin, paclitaxel, and etoposide [7]. ABCG2 was known to mediate resistance to mitoxantrone, topotecan, and irinotecan [8], and ABCC1 mediated MDR to doxorubicin and vincristine [9]. Hyper‐expression of ABCB1, ABCG2, or ABCC1 has been documented in clinical tumor specimens to mediate MDR and poor prognosis in patients bearing malignancies in breast, liver, blood, colon, and lung [10]. Therefore, ABCB1/ABCG2/ABCC1 are recognized as promising targets for the circumvention of MDR in cancer patients.

In the past two decades, many MDR modulators in specificity, such as verapamil (targeting ABCB1), MK571 (targeting ABCC1), and Ko143 (targeting ABCG2), have been reported in preclinical studies [11]. However, the clinical application of these MDR modulators to overcome drug resistance is not favorable, mainly due to adverse side‐effects, insufficient efficacy in vivo, and uncertain drug–drug interactions [12]. Besides, due to the considerable overlap of substrates for different ABC transporters, suppressing one of them alone may not be sufficient to solve MDR in clinical practice [13, 14]. To this end, broad‐spectrum inhibitors of ABC transporters are required, and only a very few have been reported. VX‐710 (biricodar), capable of inhibiting ABCB1, ABCC1, and ABCG2 simultaneously, was proved to enhance anticancer efficacy in acute myeloid leukemia, which was resistant to anticancer drug for its constitutively overexpressing the three ABC proteins [15]. More promising pan‐MDR modulators, that are more potent, less toxic, and do not display significant drug–drug interaction with anticancer drugs, should be developed to improve the efficacy of cancer therapy.

Ubiquitin specific peptidase 1, shorted as USP1, is a kind of enzyme to remove ubiquitin from proteins (DUB), which admittedly participated in regulating some pathways of damaged DNA repair, such as the Fanconi anemia (FA) pathway and translesion synthesis (TLS) process [16, 17, 18]. Increasing evidences suggested that USP1 plays vital roles in tumor pathogenesis and development [19, 20, 21], and aberrant overexpression of USP1 has been found frequently in many cancers including gastric cancer, cervical cancer, melanoma, and so on [18]. USP1 rapidly became an emerging therapeutic target of malignant tumors with the emergence of the concept “Synthetic Lethality” [22], which indicates the great prospect of USP1 inhibitors in combination therapy. Studies showed that the overexpression of USP1 induced cisplatin resistance [23], and inhibiting USP1 could attenuate cisplatin resistance [24]. What is more, inhibition of USP1 was found to potentiate the anticancer effect of other chemotherapeutic drugs in relapsed B‐cell lymphoma [25]. However, the effects of USP1 inhibitors on MDR caused by ABC transporters have not been disclosed. In this work, we first disclosed the widely potent reversing effects of KSQ‐4279, a highly‐selective USP1 inhibitor currently in clinical trial (Phase I) for advanced solid tumors (NCT05240898), on ABCB1/ABCG2/ABCC1‐induced MDR cancers, and the exact mechanisms behind were elucidated.

## 2 Results

### 2.1 KSQ‐4279 Boosted the Cytotoxic Effects of Multiple Chemo‐Drugs in ABCB1‐, ABCG2‐, and ABCC1‐Driven MDR Cancer Cells

**FIGURE 1:**
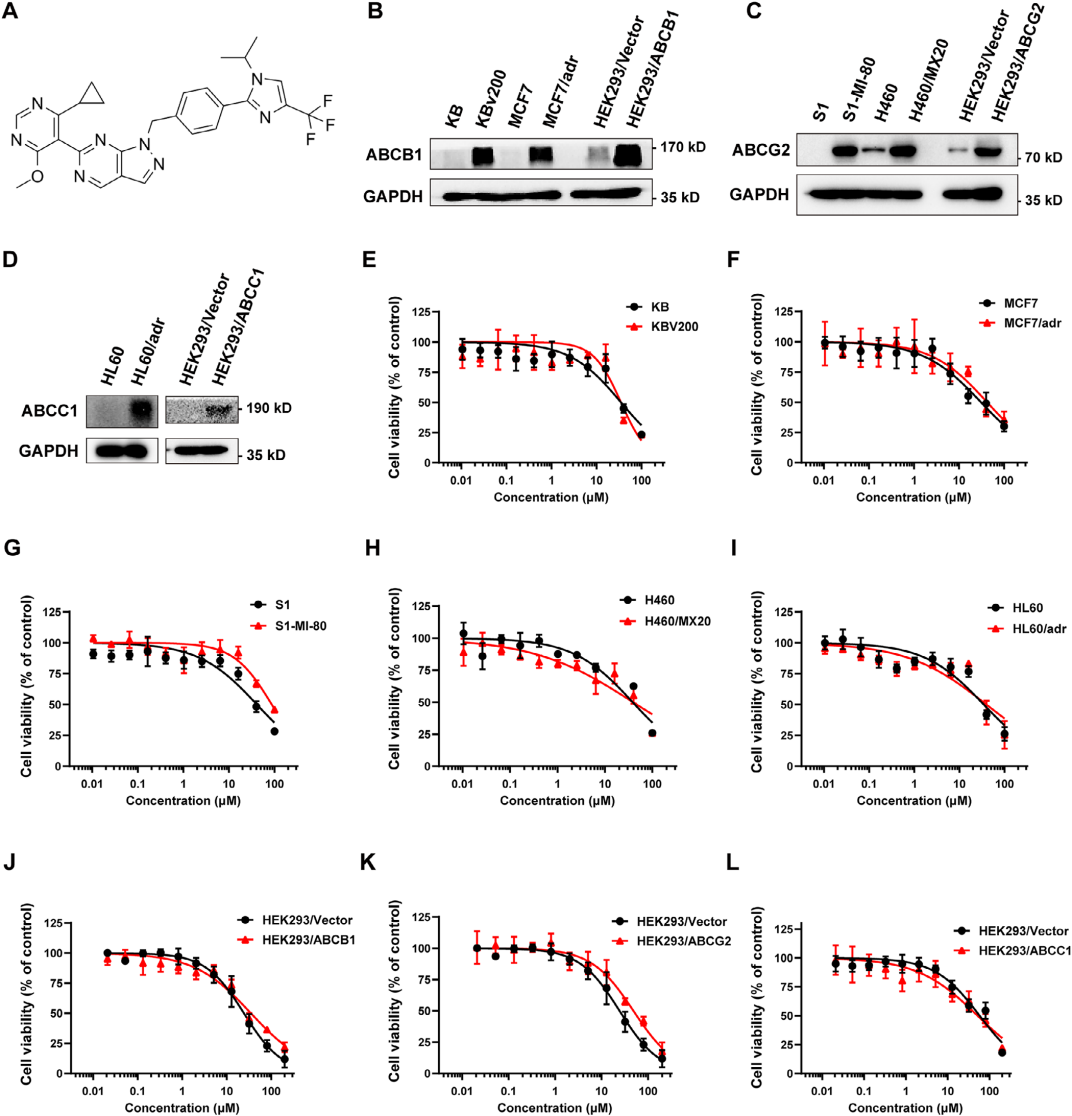
The construction and cytotoxic function of KSQ‐4279. (**A**) Chemical structure of KSQ‐4279. (**B–D**) ABCB1/ABCG2/ABCC1 proteins in the indicated MDR cells and the corresponding sensitive cells were confirmed by western blot analysis with an input control of GAPDH. (**E–L**) Survival curves for the indicated cell lines after 72‐h incubation with KSQ‐4279. Data points are reported as mean ± SD.

The chemical structure of KSQ‐4279 is shown in Figure 1A. First, the significant overexpression of ABCB1 in KBv200, MCF7/adr, and HEK293/ABCB1 cells; ABCG2 in S1‐MI‐80, H460/MX20, and HEK293/ABCG2 cells; or ABCC1 in HL60/adr and HEK293/ABCC1 cells, in comparison with their respective sensitive cells, were verified via western blot analysis (Figure 1B–D). The cytotoxicity (IC_50_) of KSQ‐4279 alone in the pairs of sensitive and MDR cell lines was tested by MTT (Figure 1E–L). KSQ‐4279 alone did not remarkably affect cell viability at the concentration < 10 µM (cells remained at > 75% viability). Therefore, in the subsequent reversal experiments, KSQ‐4279 concentrations of 2.5, 5, and 10 µM were used.

Table S1 summarized the IC_50_ values of a few chemo‐drugs in various MDR cell lines mediated by ABCB1, ABCG2, ABCC1, LRP, or MRP7 overexpression with the presence or absence of KSQ‐4279. Consistent with our data published previously, the resistant cell lines displayed different degree of MDR to the chemotherapeutic drugs compared with their parental sensitive cell lines (Table S1) [26]. In ABCB1‐overexpressing KBv200 and MCF7/adr cell lines, the antitumor effect of the ABCB1 substrate drugs (paclitaxel, vincristine [VCR], and doxorubicin [DOX]), but not the non‐substrate drugs (cisplatin) were significantly enhanced by pretreatment of KSQ‐4279. Meanwhile, KSQ‐4279 had few effects on the response of the sensitive KB and MCF7 cells to the substrate or non‐substrate drugs. Such results were also similarly obtained in the ABCG2‐hyperexpressed S1‐MI‐80 and H460/MX20 cells to mitoxantrone (MX) or topotecan and in the ABCC1‐overexpressing HL60/adr cells to doxorubicin, in contrast with their corresponding parental cells. However, KSQ‐4279 had few effects on LRP or MRP7‐mediated resistance in SW1573/2R120 and HEK293/MRP7‐2 cells. Furthermore, KSQ‐4279 was shown to strikingly improve the antitumor action of the substrate chemotherapeutic drugs in HEK293 cells with ABCB1‐stably‐transfection (HEK293/ABCB1), ABCG2‐stably‐transfection (HEK293/ABCG2), or ABCC1‐stably‐transfection (HEK293/ABCC1), but not in HEK293 cells transfected with the vector plasmid (HEK293/Vector) (Table S2).

Collectively, these data demonstrated that KSQ‐4279 could widely and significantly potentiate the chemotherapeutic cytotoxicity of traditional agents in the ABCB1‐, ABCG2‐, and ABCC1‐induced MDR tumor cells in vitro.

### 2.2 KSQ‐4279 Potentiated the Function of Chemotherapeutic Drugs In Vivo and Ex Vivo

**FIGURE 2:**
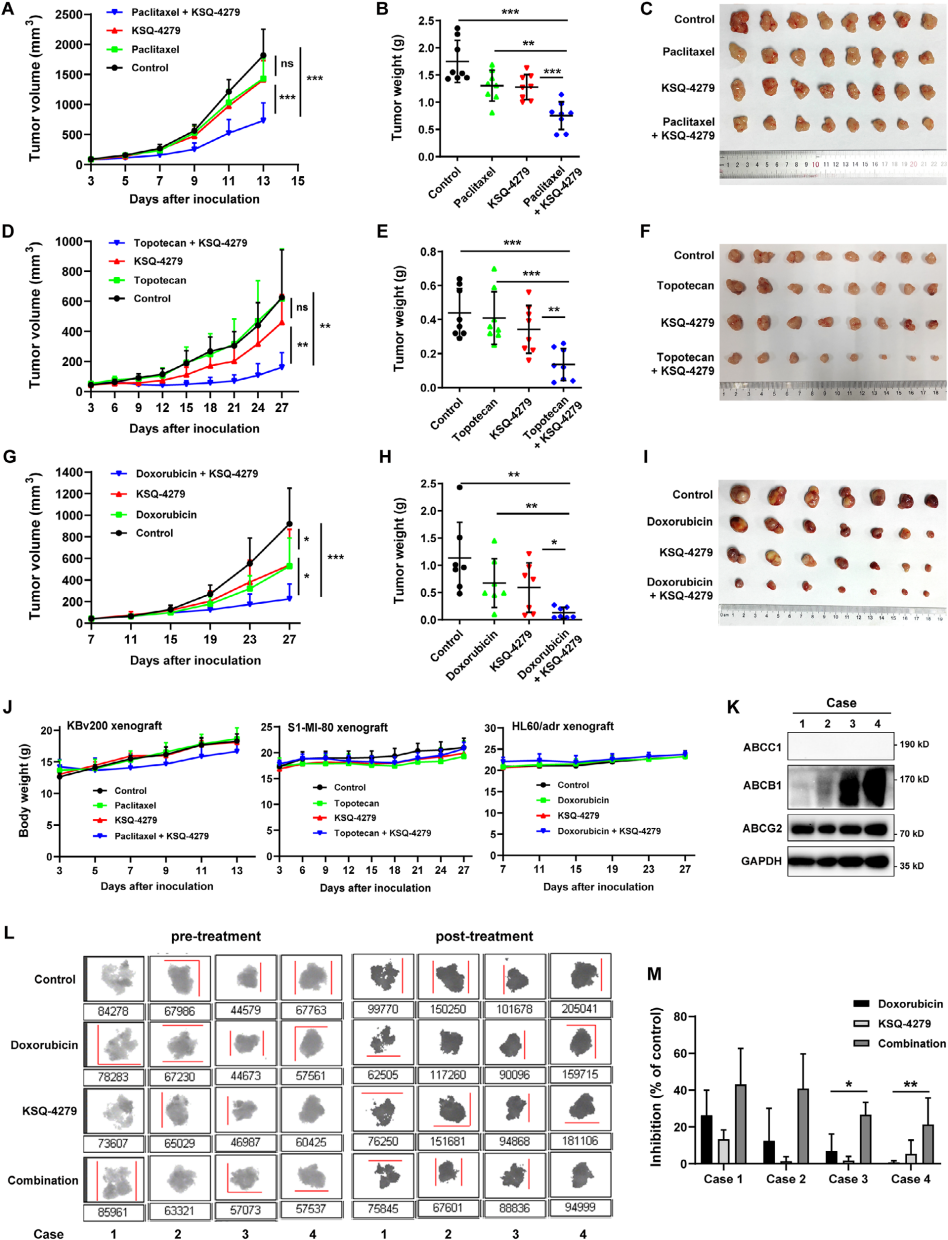
KSQ‐4279 promoted the antitumor activity of chemotherapeutic drugs in vivo and ex vivo. (**A–I**) Change of tumor volume with time and tumor weight following the indicated treatment in BALB/C nude mice bearing tumor xenograft of KBv200 (**A–C**), S1‐MI‐80 (**D–F**), or HL60/adr (**G–I**). (**J**) Change of body weight with time in tumor‐bearing mice. (**K–M**) Clinical human lung cancer specimens (cases 1–4) were used to confirm the circumvention of doxorubicin resistance by KSQ‐4279 on ex vivo. Protein expressions of ABCB1, ABCG2, and ABCC1 of the tumor specimens were analyzed via western blot (**K**), and the viability of specimens was analyzed by MTT assay and the Image Analysis System (**L–M**). Data were reported as mean ± SD (*p < 0.05; **p < 0.01; ***p < 0.001).

The phenomena of MDR reversed by KSQ‐4279 were further verified in vivo by using nude mice bearing ABCB1‐overexpressing KBv200 (Figure 2A–C), ABCG2‐overexpressing S1‐MI‐80 (Figure 2D–F), and ABCC1‐overexpressing HL60/adr (Figure 2G–I) xenografts. Compared with the saline‐control, paclitaxel (15 mg/kg) or topotecan (2 mg/kg) did not significantly inhibit tumor growth. Also, KSQ‐4279 alone (30 mg/kg) did not produce significant antitumor effect. However, the combination of KSQ‐4279 with paclitaxel or topotecan remarkably suppressed tumor growth in KBv200 or S1‐MI‐80 bearing mice (Figure 2A–F). Consistently, the combination of KSQ‐4279 also remarkably potentiated the efficacy of doxorubicin (3 mg/kg) in HL60/adr bearing mice (Figure 2G–I). Meanwhile, combination of KSQ‐4279 with these chemotherapeutic drugs did not cause apparent loss in animal body weight (Figure 2J).

Clinical human tumor specimens from lung cancer patients were used to further confirm the KSQ‐4279 combination chemotherapeutic efficacy by ex vivo experiments. Each tumor specimen was chopped and subjected to the indicated incubation (saline control, 4 µM doxorubicin, 10 µM KSQ‐4279, combination of doxorubicin with KSQ‐4279). The viability of tumor specimens was analyzed by MTT assay and an Image Analysis System (Figure 2L–M), and the expression of ABCB1, ABCG2, and ABCC1 proteins in specimens was synchronously detected via western blot assays (Figure 2K). The results showed that tumor specimens with higher ABCB1 expression (cases 3 and 4) displayed higher resistance to doxorubicin than that with lower ABCB1 expression (cases 1 and 2), which affirmed a clinical significance of ABCB1 in mediating MDR. Importantly, KSQ‐4279 combination here was shown to significantly sensitize the resistant tumor specimens to doxorubicin treatment, and a more significant enhancement in the antitumor effect was observed in the specimens with higher ABCB1 expression (Figure 2K–M).

Overall, KSQ‐4279 effectively conquered the ABCB1‐, ABCG2‐, or ABCC1‐induced MDR in vivo, and significantly relieved the ABCB1‐mediated MDR in clinical lung cancer patients’ ex vivo specimen models, which encouraged further study in combination of KSQ‐4279 and chemotherapy in the patients with MDR cancer in clinic.

### 2.3 KSQ‐4279 Augmented Cellular Retention of Doxorubicin in ABCB1‐, ABCG2‐, and ABCC1‐Diven MDR Cancer Cells

**FIGURE 3:**
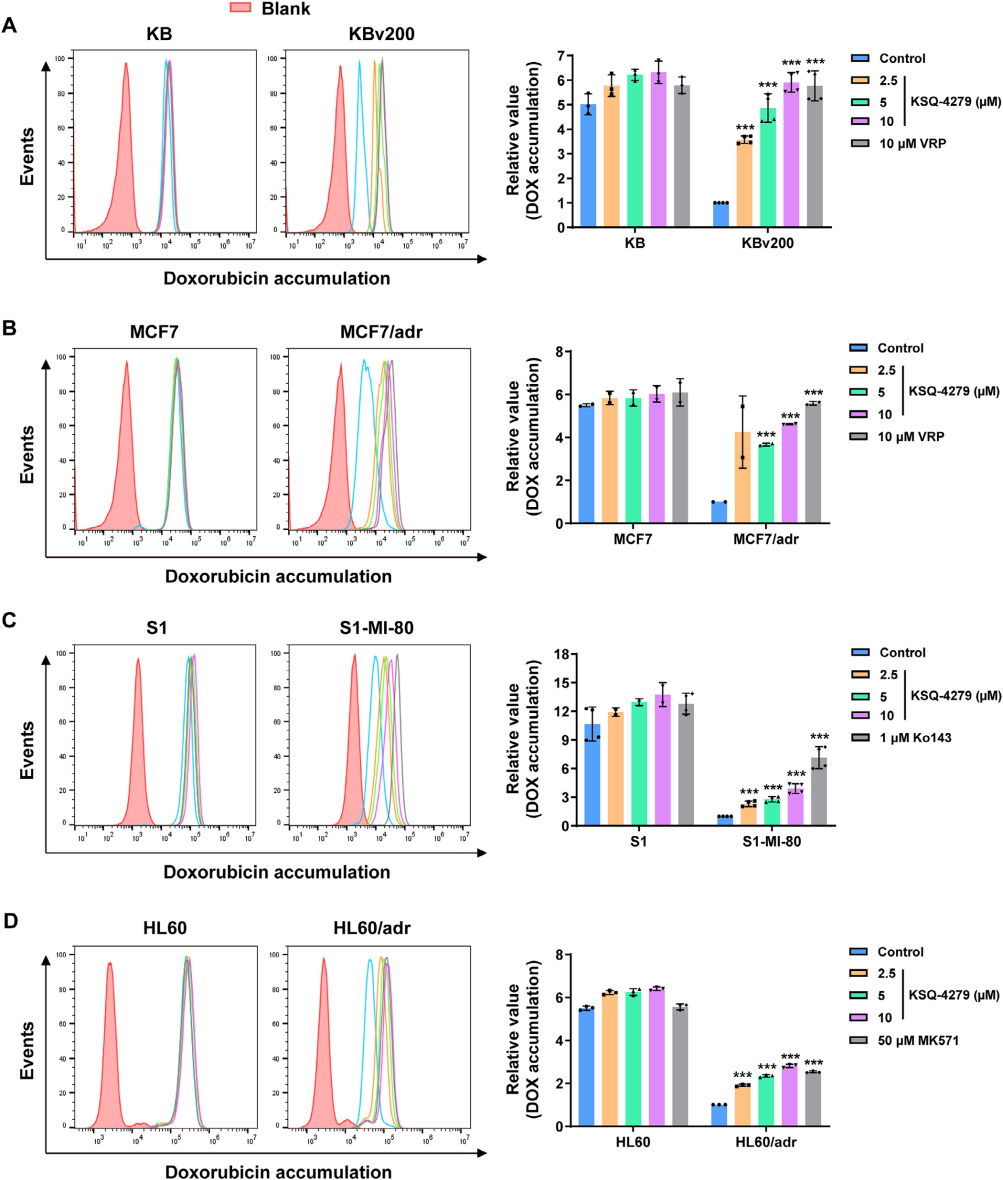
Effect of KSQ‐4279 on intracellular remains of doxorubicin. (**A–D**) Left: The fluorescent doxorubicin reserved in ABCB1‐overexpressing MDR cells (KBv200, MCF‐7/adr), ABCG2‐overexpressing MDR S1‐MI‐80, and ABCC1‐overexpressing MDR HL60/adr cells, and the corresponding parental cells were detected using flow cytometric assay. Right: Bar graphs summarizing the relative doxorubicin accumulation from the cells. Normalized the final value by comparing to the fluorescence of MDR control cells. Data were reported as mean ± SD (*p < 0.05; **p < 0.01; ***p < 0.001).

For elucidating the mechanisms contributing to the circumvention of MDR by KSQ‐4279, cellular retention of chemotherapeutic drug (doxorubicin) was detected via flow cytometry. Similar to the positive controls of verapamil (VRP, a definite ABCB1 inhibitor), Ko143 (a definite ABCG2 inhibitor), and MK571 (a definite ABCC1 inhibitor), pretreatment of KSQ‐4279, in a dose dependent manner, notably elevated the cellular reservation of doxorubicin in ABCB1‐, ABCG2‐, and ABCC1‐overexpressing MDR cells (KBv200, MCF7/adr, S1‐MI‐80, HL60/adr) (Figure 3A–D). However, the pretreatment of KSQ‐4279 did not appreciably affect doxorubicin accumulation in the corresponding sensitive cells (KB, MCF7, S1, and HL60).

These data indicated KSQ‐4279 as an effective MDR modulator capable of increasing the cellular storage of chemotherapeutic drugs in ABCB1/ABCG2/ABCC1 highly expressed MDR cancer cells.

### 2.4 KSQ‐4279 Reduced Doxorubicin Efflux Mediated by ABCB1/ABCG2/ABCC1

**FIGURE 4:**
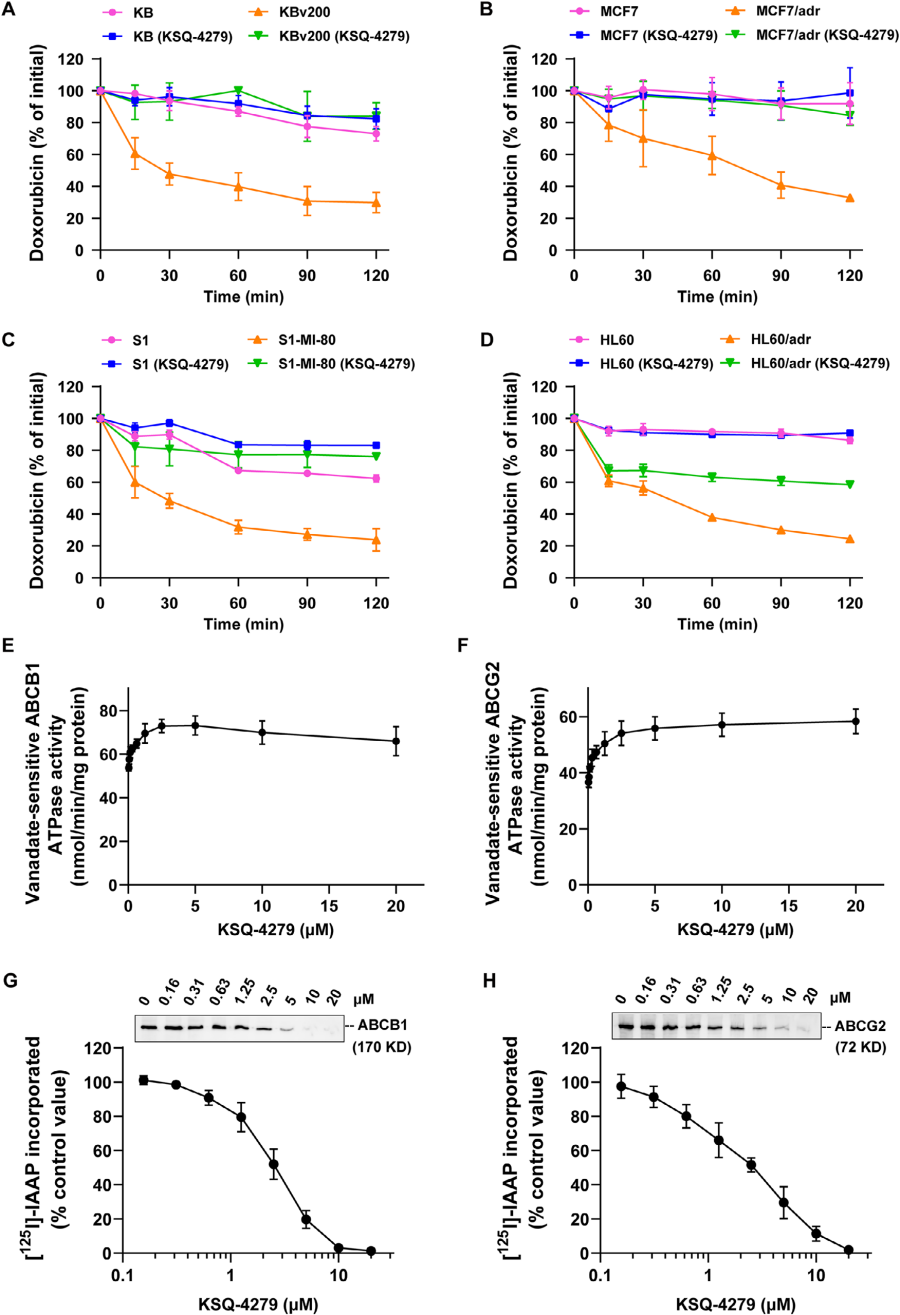
KSQ‐4279 blocked doxorubicin efflux, provoked ATPase activity, and restrained the [^125^I]‐IAAP labeling of ABCB1/ABCG2. (**A–D**) The remaining doxorubicin in cells was detected by flow cytometry at different time points in the drug efflux experiments. (**E–F**) The change of ABCB1/ABCG2 ATPase activity (which sensitive to vanadate) caused by concentrations of KSQ‐4279. (**G–H**) KSQ‐4279 scrambled for photolabeling of ABCB1/ABCG2 with [^125^I]‐IAAP. Data were reported as mean ± SD.

Given the pump‐like function of ABC transporters, the impact of KSQ‐4279 on ABCB1‐, ABCG2‐, and ABCC1‐caused doxorubicin efflux was further evaluated by quantifying the change of doxorubicin inside cancer cells over time (Figure 4A–D). Results showed that, after an initial 3‐h incubation with doxorubicin (10 µM) and the replacement of drug‐free medium, the intracellular doxorubicin content declined gradually both in sensitive and resistant/MDR cells, and the drug efflux rate in MDR cells was unsurprisingly much faster than that in the corresponding sensitive cells. Importantly, KSQ‐4279 (10 µM) remarkably inhibited the rate of doxorubicin efflux in the MDR cells. These results supported the aforementioned increase in doxorubicin accumulation and circumvention of ABCB1/ABCG2/ABCC1‐induced MDR by KSQ‐4279.

Since the efflux function is closely relevant to energy supply, the ATP hydrolysis of ABC transporters affected by KSQ‐4279 was further measured. As shown in Figure 4E,F, when low dose of KSQ‐4279 (< 5 µM) was given, both ATPases of ABCB1 and ABCG2 were activated following an increase in KSQ‐4279 concentration. When the KSQ‐4279 was given over 10 µM, the rising activity of ABCB1 and ABCG2 ATPases got into a plateau, indicating KSQ‐4279 might interact with the substrate‐binding area of ABCB1/ABCG2 and promote ATP to hydrolyze. [^125^I]‐IAAP, the photo‐affinity analogue of prazosin, is demonstrated as a substrate transported by both ABCB1 and ABCG2 proteins, and other ABCB1/ABCG2 substrates or inhibitors are known to struggle for photolabeling of transporters with [^125^I]‐IAAP [27]. In order to confirm how KSQ‐4279 interacted with the pockets of ABCB1 and ABCG2 proteins, ABCB1‐ or ABCG2‐expressed crude membranes, derived from High Five insect cells, were treated by [^125^I]‐IAAP and different doses of KSQ‐4279. The results showed that KSQ‐4279 concentration‐dependently restrained the [^125^I]‐IAAP photolabeling of both ABCB1 and ABCG2, and the concentrations suppressed 50% photolabeling were both around 2.5 µM (Figure 4G,H). Therefore, KSQ‐4279 might competitively occupy the substrate‐binding domains of ABCB1/ABCG2, resulting in the reduction in substrate drugs’ efflux.

### 2.5 KSQ‐4279 Had No Effects on the Expression and Localization of ABCB1, ABCG2, and ABCC1 in MDR Cells

**FIGURE 5:**
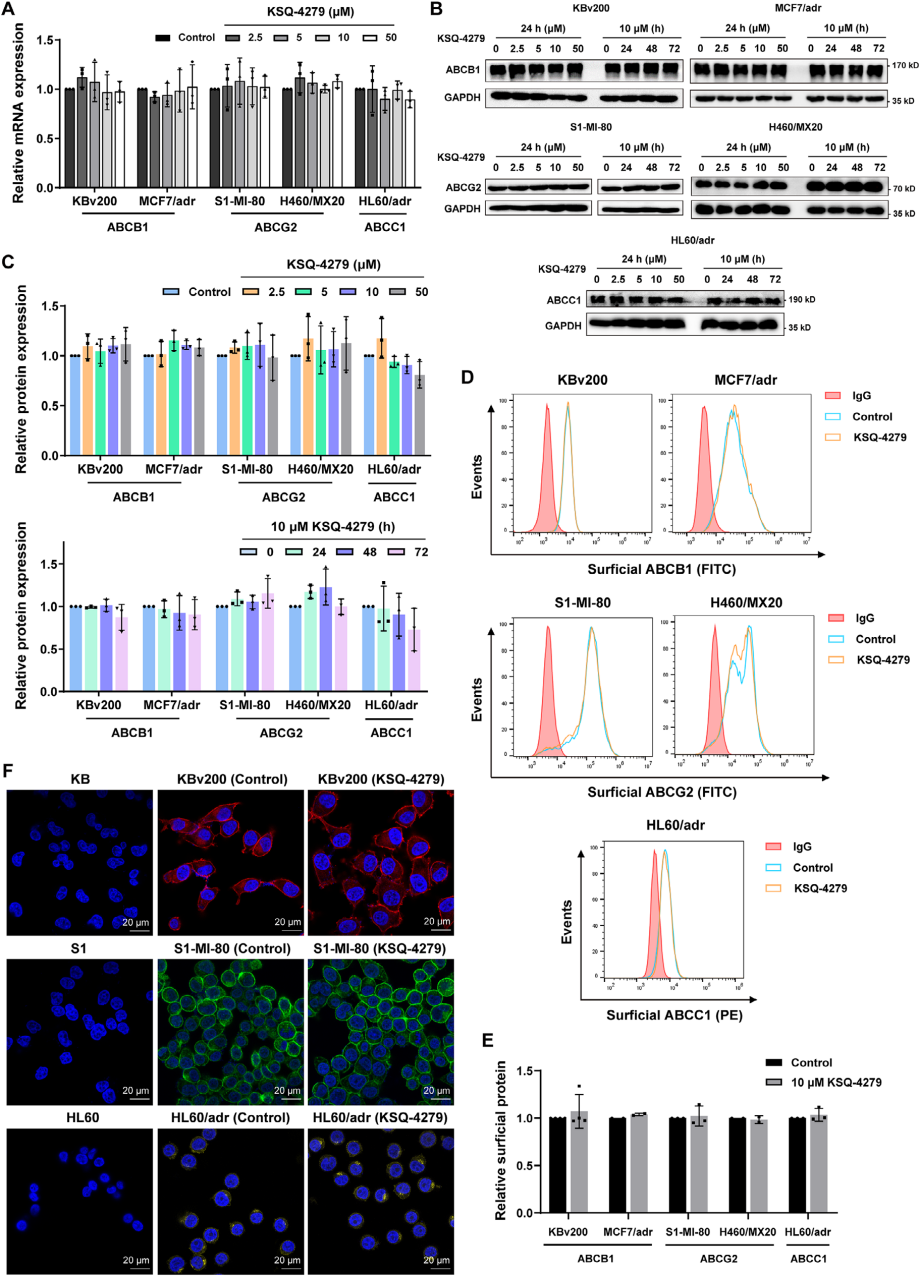
Effects of KSQ‐4279 on ABCB1/ABCG2/ABCC1 expression and subcellular localization. (**A**) ABCB1/ABCG2/ABCC1 mRNA was tested by qRT‐PCR. (**B**) ABCB1/ABCG2/ABCC1 proteins in indicated cells treated by different doses of KSQ‐4279 and for different time were assessed via western blot with the GAPDH as an input control. (**C**) Bar graphs showing the relative protein expression compared with the no treatment control from the western blot data in (B). (**D**) KSQ‐4279 had few effects on the ABCB1/ABCG2/ABCC1 protein enriched at cell surface according to flow cytometric analysis. (**E**) Quantification for (D). (**F**) Subcellular localization of ABCB1/ABCG2/ABCC1 protein was visualized in the indicated cell lines by the Confocal Microscope (Laser) with 63× oil lens (blue: nuclei labeled by DAPI, red: ABCB1, green: ABCG2, yellow: ABCC1). Data were reported as mean ± SD.

Apart from the drug efflux activity of ABC transporters, their protein expression is another crucial factor regulating the drug sensitivity of MDR tumors. The changes in ABCB1, ABCG2, and ABCC1 expression following KSQ‐4279 incubation was revealed by qRT‐PCR assays and western blot analysis. In all MDR cancer cell lines tested, incubation of KSQ‐4279 had few effects on ABCB1/ABCG2/ABCC1 expression both in mRNA level (Figure 5A) and protein level (Figure 5B,C), even given the treated concentration of KSQ‐4279 higher to 50 µM or the incubated time longer to 72 h. Besides, since the amounts of ABC transporters on cell membrane matter a lot to the overall drug‐transporting activity, the expressions of ABCB1, ABCG2, and ABCC1 in the surface of MDR cancer cells were detected by flow cytometry, with the results of no significant alteration caused by KSQ‐4279 incubation (Figure 5D,E). Moreover, localization of ABCB1, ABCG2, or ABCC1 in cells was also visualized by immunofluorescence using confocal microscopy (Figure 5F). Consistent with results of Figure 1B,C, there was higher expression of the transporters in the MDR cells (ABCB1 on KBv200, ABCG2 on S1‐MI‐80, and ABCC1 on HL60/adr) compared with the parental KB, S1, and HL60 cells. Importantly, KSQ‐4279 made no impact on the localization and expression level of all three transporter proteins. These results indicated that KSQ‐4279 reversed the pan‐ABC transporters‐caused MDR mainly through discouraging the pump‐like activity of ABCB1, ABCG2, and ABCC1, rather than altering their expression level or cellular localization.

### 2.6 MDR Reversal by KSQ‐4279 Did Not Rely on Blocking AKT/ERK Signaling

**FIGURE 6:**
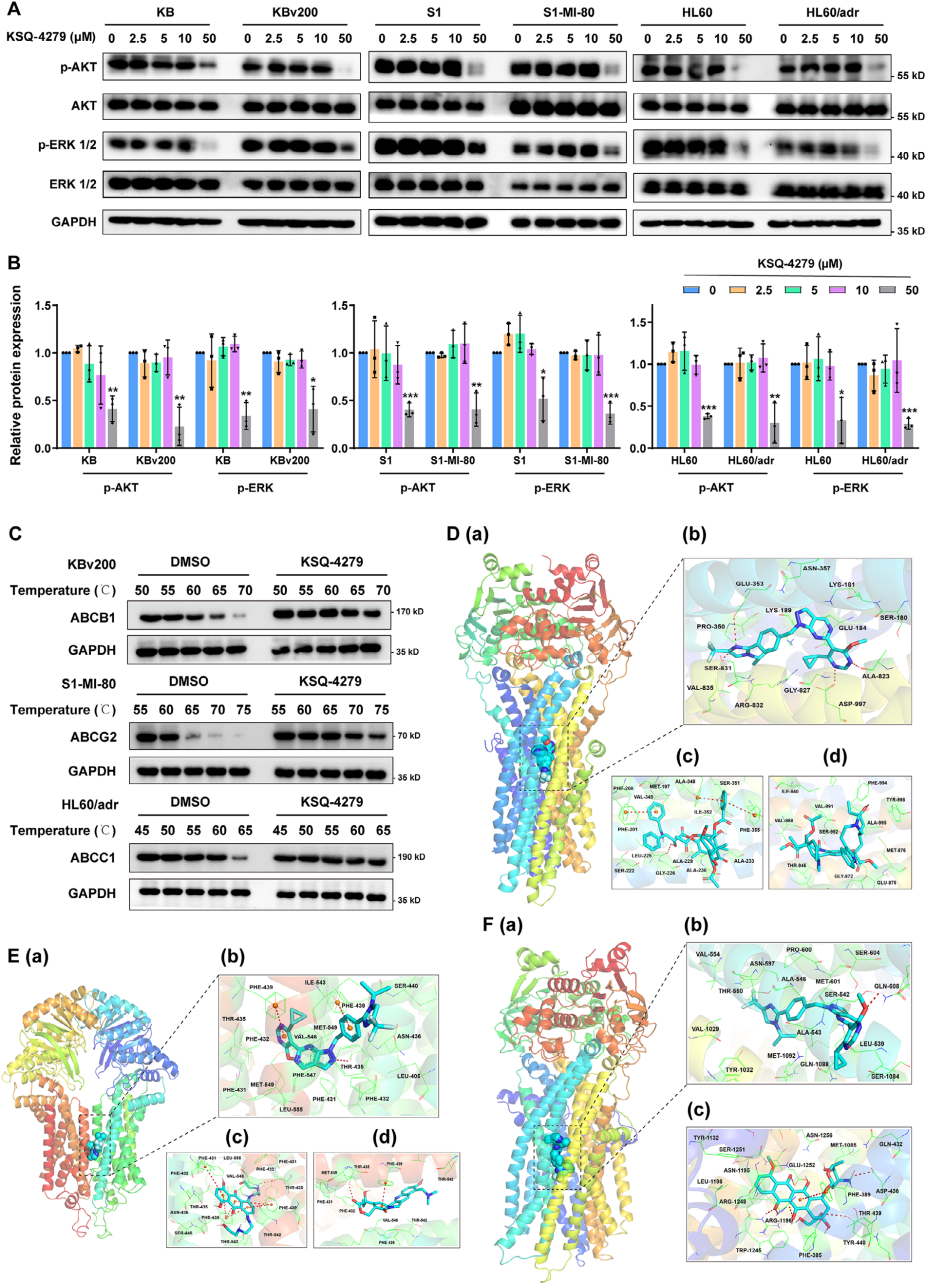
Influence of KSQ‐4279 on AKT and ERK transduction and analysis of KSQ‐4279 binding with ABCB1/ABCG2/ABCC1. (**A**) Western blot showed the action of KSQ‐4279 on AKT and ERK transduction. GAPDH was as an input control. (**B**) Bar graphs for the relative protein expression of the various signaling molecules detected in (A). Data were reported as mean ± SD (*p < 0.05; **p < 0.01; ***p < 0.001). (**C**) Binding of KSQ‐4279 with ABCB1, ABCG2, and ABCC1 were assessed by CETSAs. Aliquots of cell lysates incubated with DMSO or KSQ‐4279 (10 µM) were heated at indicated temperatures, and the presence of KSQ‐4279‐bound ABCB1/ABCG2/ABCC1 proteins was analyzed via western blots. (**D**) Molecular docking of (a and b) KSQ‐4279, (c) paclitaxel, and (d) vincristine with to substrate‐binding region of ABCB1. (**E**) Molecular docking of (a and b) KSQ‐4279, (c) mitoxantrone, and (d) topotecan to the substrate‐binding area of ABCG2. (**F**) Molecular docking of (a and b) KSQ‐4279, and (c) doxorubicin to the substrate‐binding domain of ABCC1. Molecular 3D structures were obtained from the web of PubChem (https://pubchem.ncbi.nlm.nih.gov/), and the crystal structures of ABC transporters were obtained from the web of Protein Data Bank (https://www.rcsb.org/). The docking was performed by using software of AutoDock Vina.

Since USP1 is a deubiquitinase that functions as stabilizing the downstream proteins, the possibility of KSQ‐4279 directly decreasing ABCB1/ABCG2/ABCC1 proteins via suppressing USP1 could be ruled out based on the results of Figure 5B–D, but whether the MDR reversal effects mediated by KSQ‐4279 depend on the secondary effects of USP1 inhibition need to be confirmed. PI3K/AKT pathway and MEK/ERK1/2 pathway were previously reported as crucial regulators of MDR mediated by ABC transporters [28, 29, 30, 31, 32], while recent studies showed that USP1 inhibition caused repression of AKT and ERK signaling [24, 33, 34]; thus, the influence of KSQ‐4279 on AKT and ERK transduction was further assessed via western blot assays. The results displayed that KSQ‐4279 significantly decreased AKT/ERK phosphorylation at a high concentration of 50 µM, but did not affect AKT and ERK signaling at the practical MDR reversal doses of 2.5, 5, or 10 µM (Figure 6A,B), which suggest that MDR reversed by KSQ‐4279 was independent of the secondary effect of USP1 inhibiting AKT or ERK signaling.

### 2.7 Binding of KSQ‐4279 With ABCB1, ABCG2, and ABCC1

Considering the conclusion of Figure 4E–H that KSQ‐4279 might competitively occupy the substrate‐binding domains of ABCB1/ABCG2, we performed Cellular Thermal Shift Assay (CETSA) to confirm the direct binding of KSQ‐4279 with ABCB1, ABCG2, and ABCC1. As shown in Figure 6C, the incubation of KSQ‐4279 significantly enhanced the thermal stability of ABCB1, ABCG2, and ABCC1 proteins, proving the binding of KSQ‐4279 with these three ABC transporters. Then the binding modes were further analyzed through molecular docking, and results showed that KSQ‐4279 could occupy the drug‐binding sites of ABCB1, ABCG2, or ABCC1, respectively, with the lowest binding energy of −9.2 kcal/mol (Figure 6D(a,b)), −11.7 kcal/mol (Figure 6E(a,b)), and −9.8 kcal/mol (Figure 6F(a,b)). Strikingly, compared with the binding energy of a few conventional ABC transporter substrate drugs (e.g. ABCB1 substrates: paclitaxel [Figure 6D(c), −8.9 kcal/mol] and vincristine [Figure 6D(d), −7.0 kcal/mol]; ABCG2 substrates: mitoxantrone [Figure 6E(c), −8.4 kcal/mol] and topotecan [Figure 6E(d), −9.8 kcal/mol]; and ABCC1 substrate: doxorubicin [Figure 6F(c), −9.5 kcal/mol]), KSQ‐4279 appeared to exhibit a higher binding affinity for all three MDR transporters. Specific interactions of ABC transporter residues with these ligands have been presented in Figure S1A–H by 2D drawings, in which KSQ‐4279 seemed to interact with ABCB1/ABCG2/ABCC1 in a more diverse and balanced binding mode. These data suggested the potentially preferential binding of ABCB1/ABCG2/ABCC1 to KSQ‐4279, which also supported the conclusion of Figure 4E–H, and further explained the MDR reversal by KSQ‐4279 at the structural level.

## 3 Discussion

MDR widely observed in various cancer types is an unresolved medical problem hindering effective chemotherapy. Highly expressed ABC transporters in resistant/refractory carcinomas are considered the most important contributing factor causing MDR [35, 36, 37]. Under physiological condition, ABC transporters mainly maintain a homeostasis of endogenous or exogenous substances in cells by regulating their secretion and excretion. But in cancer cells, abundant ABC transporters effectively protect the cells from chemotherapeutic cytotoxicity by letting drugs out, thereby bringing about MDR and bad prognosis [38]. The inhibition of ABC transporters‐induced drug efflux is accepted as a hopeful strategy to conquer MDR.

ABCB1, ABCG2, and ABCC1 are the three considerable drug transporters mediating MDR [39, 40, 41], and numerous relevant modulators have been studied for their potential to reduce MDR in vitro and in vivo. These ABC transporter modulators are generally classified into four generations according to their functional characteristics [42]: (a) the first generation (for instance, tamoxifen, quinidine, cyclosporin A, and verapamil) worked in vitro but produced disappointing treatment outcomes in vivo due to awful toxicity [35, 43]. (b) The second generation (such as biricodar, gallopamil, valspodar, S97882, and PSC833) was proved effective and less toxic both in vitro and in vivo. However, they gave rise to some unpredictable side effects in clinic due to pharmacokinetic drug–drug interaction with the traditional chemotherapeutic agents [44, 45]. (c) The third generation (such as zosuquidar, laniquidar, XR9576, and GF120918) exhibited significantly less pharmacokinetic drug–drug interactions. They are well tolerated in clinical applications and represent promising candidates for MDR reversal in cancer patients [46, 47, 48]. (d) The fourth generation (such as curcumin and neochamaejasmin B) demonstrated better oral bioavailability and favorable safety profile. They were required to keep off MDR by directly reducing ABC transporters expression [49, 50]. Although remarkable advancement has been made during the exploration and development of MDR modulators, none of them have been clinically approved for MDR circumvention. The discovery and development of novel and potent MDR modulators is still in urgency.

KSQ‐4279 is a defined first‐in‐class USP1 inhibitor under clinical development (NCT05240898). Displaying good bioavailability and excellent safety profile, KSQ‐4279 represents a highly promising anticancer agent when used alone or in combination for treating various cancers [51]. In this study, the innovative application of KSQ‐4279 as an MDR modulator was investigated. KSQ‐4279 was shown to significantly elevate the antitumor function of multiple conventional chemotherapeutic drugs in MDR cancer cell lines driven by ABCB1/ABCG2/ABCC1 overexpression in vitro independently on its own cytotoxicity (Tables S1 and S2 and Figure 1). The potent efficacy of KSQ‐4279 in reversing MDR was further confirmed in vivo by MDR tumor xenografts bearing mice, without causing additional toxicity (Figure 2A–J). Moreover, KSQ‐4279 was also validated to potentiate the antitumor ability of doxorubicin (a substrate drug for multiple ABC transporters) in primary culture of tumor specimens obtained from drug‐refractory lung cancer patients ex vivo (Figure 2K–M). These data suggested KSQ‐4279 in combination with conventional chemotherapy is a promising therapeutic avenue for clinically refractory MDR. Considering various failures of previous MDR modulators, more information should be illuminated in the future, for example, the detailed long‐term efficacy and toxicity profiles about organ‐specific toxicity, maximum tolerated dose, and pharmacokinetic/pharmacodynamic (PK/PD) data, which is essential to establish its safety and inform dosing regimens for KSQ‐4279 combination therapies. Besides, for the generalizability of the ex vivo findings, huge amounts of patient‐derived organoids overexpressing ABCG2 and ABCC1 need to be collected and tested, which would strikingly strengthen the translational relevance of KSQ‐4279 as a pan‐MDR modulator.

Mechanistically, using flow cytometric analysis, we verified KSQ‐4279 could restrain the drug efflux activities of ABCB1/ABCG2/ABCC1 (Figure 4A–D), thus remarkably making more drugs arrested in cells (Figure 3). Furthermore, data from the vanadate‐sensitive ATPase assays (Figure 4E,F) and the [^125^I]‐IAAP‐photolabeling assays (Figure 4G,H) showed that KSQ‐4279 inhibited substrate drugs interacting with ABCB1/ABCG2 in a competitive manner, which was evidenced via CETSA (Figure 6C) and molecular docking (Figure 6D–F). In addition, by experiments of qRT‐PCR, western blot, flow cytometry, and immunofluorescence, at the practical MDR reversal doses, KSQ‐4279 was proved to neither decrease ABCB1/ABCG2/ABCC1 expression nor reduce their enrichment in the cell membrane (Figure 5). These characteristics of KSQ‐4279 indicated it could be classified into the third‐generation MDR modulator with a promising prospect of development. The circumvention of MDR by KSQ‐4279 has also been confirmed independent of the potential secondary effects of USP1 inhibition (Figure 6A,B), which hinted ABCB1, ABCG2, and ABCC1 might be the new targets of KSQ‐4279.

Another salient point of this work is that KSQ‐4279 was shown to simultaneously conquer ABCB1‐, ABCG2‐, and ABCC1‐driven MDR. Due to the considerable overlap of substrates for different ABC transporters, suppressing one of them alone may not be sufficient to solve MDR in clinical practice [13, 14]. Our findings advocate the rational application of KSQ‐4279 as a pan‐MDR modulator to strengthen the function of conventional chemotherapeutic agents in tumors with the major ABC transporters simultaneously overexpressed in the clinical setting. As ABCB1/ABCG2/ABCC1 also plays crucial physiological roles in protecting normal tissues from xenobiotics and toxic metabolites, inhibition of these transporters may result in some unpredictable toxicity, and clinical dosing regimens for MDR modulators combination therapies should be taken with the utmost care.

**FIGURE 7:**
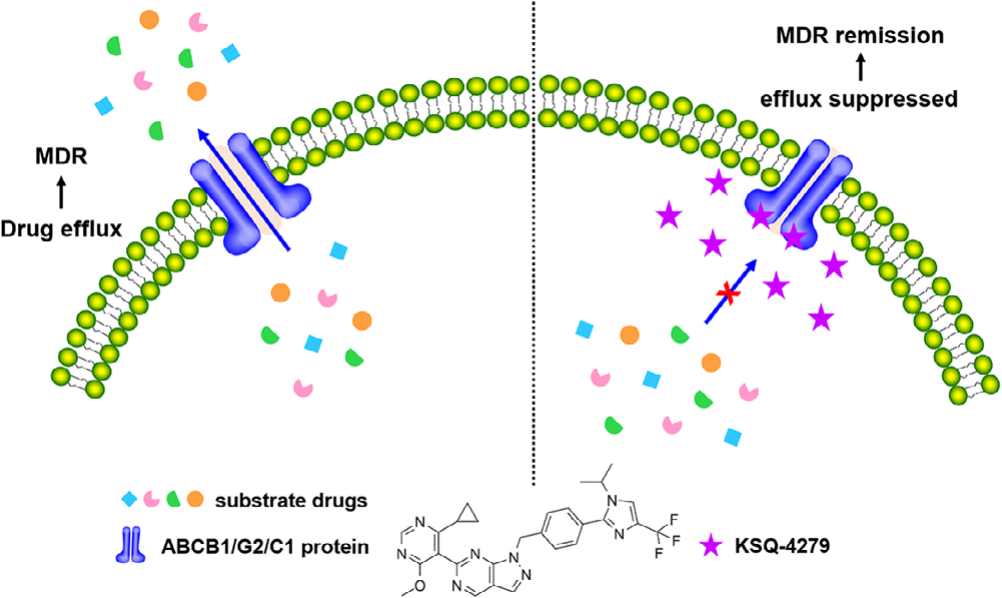
KSQ‐4279 competitively takes up the drug‐binding areas (TMD) of ABCB1/ABCG2/ABCC1 to inhibit efflux of the substrate drugs, thereby raising the intracellular reservation of the drugs and circumventing MDR.

Collectively, our data demonstrated that KSQ‐4279 could overcome ABCB1‐, ABCG2‐, and ABCC1‐induced MDR simultaneously by taking up the substrate‐binding sites of the transporters in a competitive manner and blocking their drug‐efflux capability (Figure 7). This is the first study investigating the unique combination of USP1 inhibitor (KSQ‐4279) and traditional chemotherapy to overcome MDR, which disclosed ABCB1, ABCG2, and ABCC1 as the new targets of KSQ‐4279 independent of its acknowledged target USP1, and hinted a novel combination strategy in clinic to treat cancer patients bearing drug‐refractory tumors.

## 4 Material and Methods

### 4.1 Reagents

KSQ‐4279 (C‐1550) was supplied by Chemgood (Henrico, VA, USA). Doxorubicin (HY‐15142A), paclitaxel (HY‐B0015), vincristine (HY‐N0488), topotecan (HY‐13768), and verapamil (HY‐14275) were from supplier of MedChem Express (Monmouth Junction, NJ, USA). Cisplatin (S1166), Ko143 (S7043), mitoxantrone (S2485), and MTT reagent (S6821) were acquired from Selleck Chemicals (Houston, TX, USA). DMSO, Tween‐80, Tween‐20, and PEG300 were supplied by Sigma‐Aldrich (St. Louis, MO, USA). Antibody for human ABCB1 (22336‐1‐AP), GAPDH (60004‐1), AKT (60203‐2), p‐AKT (80455‐1), ERK1/2 (28733‐1‐AP), and p‐ERK1/2 (28733‐1‐AP) were supplied by Proteintech Group, Inc. Antibody for human ABCG2 (#AB40537) was bought from Abscitech (Baltimore, USA). Human ABCC1 antibodies (sc‐18835) was obtained from Santa Cruz Biotechnology (Santa Cruz, CA, USA). Penicillin‐streptomycin solution, trypsin‐EDTA, DMEM, and RPMI‐1640 medium were supplied by Gibco (Grand Island, NY, USA). Fetal bovine serum (FBS) was supplied by ExCell Bio. Regent Kits about RNA‐purification, reverse transcription, and qPCR were acquired from EZBioscience (Roseville, MN, USA).

### 4.2 Cell Lines and Cell Culture

Human cell line KB (oral carcinoma) and its MDR counterpart KBv200 (ABCB1‐overexpressing), leukemia HL60, and its MDR counterpart HL60/adr (ABCC1‐overexpressing) were kindly provided by Dr. Xu‐Yi Liu (Cancer Hospital of Beijing, Beijing, China), and were cultivated in RPMI‐1640 replenished with penicillin‐streptomycin (1%) and FBS (10%). Cell lines of MCF‐7 (human breast cancer) and its MDR‐line MCF‐7/adr (ABCB1‐overexpressing), colon cancer S1 and its MDR counterpart S1‐MI‐80 (ABCG2‐overexpressing), lung squamous cancer SW1573, and its LRP‐overexpressing MDR cell line SW1573/2R120, and HEK293 and its ABCB1/ABCG2‐482‐R2/ABCC1/MRP7/Vector stable‐transfected lines [52] were generously provided by Dr. Susan Bates (Columbia University/New York Presbyterian Hospital, Manhattan, NY, USA), and were cultured in the DMEM added with penicillin‐streptomycin (1%) and FBS (10%). All cells were nursed in the atmosphere (37°C, 5% CO_2_) for a normal growth.

### 4.3 Assessing Cytotoxic Effects and MDR Reversal Function Through MTT Assay

Note that 2000–3000 cancer cells were grown in the 96‐well plates and treated by the desired concentration range of KSQ‐4279 for 72 h. Then, 10% 5 mg/mL MTT regent was append to the medium of cells for incubation additional 4 h at 37°C. Afterward, the supernatant was thrown away, and the dark blue crystal was dissolved by DMSO (150 µL). Lastly, the absorbance was recorded at 570/630 nm.

For estimating the effects of KSQ‐4279 on reversing MDR, tumor cells were pretreated by positive drugs (as verapamil, Ko143, MK571) or KSQ‐4279 first, followed by addition of different concentrations of chemotherapeutic drugs under investigation. The cell viability after treatment with the drug combination was measured by MTT assay stated hereinabove.

### 4.4 Animal Experiments

Animal experiments involved in this research was approved by the Animal Ethics Committee of Sun Yat‐sen University Cancer Center (L102042023100C), and all principles of Declaration of Helsinki have been rigorously followed.

The animal work was carried out following our established protocol reported in our previous publication [53]. Briefly, suspension of ABCB1‐, ABCG2‐, or ABCC1‐driven MDR cell lines (3.5 × 10^6^ KBv200 cells, 1 × 10^7^ S1‐MI‐80 cells, or 2 × 10^7^ HL60/adr cells in 200 µL PBS) were planted into the flank subcutaneous layer of female nude mice. When the tumor xenografts have grown to approximately 100 mm^3^, the mice were divided randomly into four groups with treatments described here: (a) saline; (b) intraperitoneal injecting paclitaxel (15 mg/kg), topotecan (2 mg/kg), or doxorubicin (3 mg/kg); (c) intragastric feeding KSQ‐4279 (30 mg/kg). Then, dissolved in DMSO first, and appended 4.5 times of saline, and four times of PEG300 and half of Tween‐80; and (d) combination of KSQ‐4279 and paclitaxel/topotecan/doxorubicin. The treatment was administered every other day and the changes of body weight and tumor volume were followed up in the desired time. Tumor volume (*V*) was calculated by the algorithm of “*V* = length × width^2^ /2.” When the average volume of tumors treated by saline nearly to 2000 mm^3^, all mice were humanely killed, and all tumor xenografts were taken out for photographing and weighing.

### 4.5 Ex Vivo Experiments

For appraising the MDR reversal efficacy of KSQ‐4279 ex vivo, histoculture drug response assays were conducted in tumor specimens collected from lung cancer patients using the method described previously [54]. The ethical approval for collection and use of patient‐derived tumor specimens was granted by Sun Yat‐sen University Cancer Center (approval number: B2024‐406‐01). Briefly, lung cancer specimens resected from patients within 6 h were collected and proceed to the processing described here: first, dissecting the specimens into small pieces (almost 1 mm^3^); second, culturing the primary tumor pieces on the filter‐papers scaffolded in 24‐well plates with 1.5 mL DMEM medium; third, tumor response (72‐h treatment) to the following treatment groups were evaluated by an Image Analysis System: (a) saline (as control), (b) doxorubicin (4 µM), (c) KSQ‐4279 (10 µM), or (d) the combination of KSQ‐4279 (10 µM) and doxorubicin (4 µM). The tumor mass was stained with MTT reagent and the images were captured by an imaging machine; finally, the tumor growth inhibition rate (IR) was measured by using algorithm of “IR (%) = [1 − (BA/*A*)_drug‐treatment_/(BA/*A*)_control_] × 100.” (Herein, *A* (area) refers to the area of the tumor tissue before MTT staining, and BA (blue area) refers to the intensity of the stained area after addition of MTT reagent.)

### 4.6 Cellular Accumulation of Doxorubicin and Drug Efflux Assays

For assessing the action of KSQ‐4279 on doxorubicin reservation in MDR cells, cells were preincubated with desired KSQ‐4279 or DMSO for 3 h, followed by additional 3‐h incubation with doxorubicin (10 µM). Then, collecting all cells and washing, cells were resuspended in PBS. The cellular doxorubicin was determined by flow cytometry (excitation: 475–485 nm; emission: 575–585 nm).

To investigate how KSQ‐4279 affects the drug discharge, MDR cells were preincubated with doxorubicin (10 µM) for 3 h, then washed the epibiotic doxorubicin off with PBS. Afterward, incubating cells in fresh medium with KSQ‐4279 (10 µM) or DMSO at 37°C for the designated time points. Finally, measuring the amount of residual doxorubicin in cells by flow cytometry as described above.

### 4.7 ATPase Assay

Colorimetric ATPase activity assays were conducted by consulting to the method established previously [55]. Solution preparation process was as follows: (a) buffer for ATPase assay (pH 6.8; mixture of 50 mM KCl, 5 mM sodium azide, 2 mM EDTA, 1 mM DTT, and 10 mM MgCl_2_); (b) detection reagent (a mixture of 35 mM ammonium molybdate, 10% ascorbic acid, and 15 mM zinc acetate). Crude membranes (100 µg protein/mL), which were derived from the ABCB1‐expressed (or ABCG2‐expressed) High Five insect cells, were treated at 37°C by gradient‐diluted KSQ‐4279 in company with 0.3 mM sodium orthovanadate (Na_3_VO_4_) (or 1.2 mM for ABCG2) in the assay buffer for 5 min. Then the reaction of ATP hydrolysis was initiated by incubating with Mg‐ATP (5 mM) for 20 min (or 10 min for ABCG2), followed by terminating reaction with 10% SDS solution (30 µL). Additionally, incubating with the detection reagent for 20 min at 37°C and detecting the absorbance at 750 nm by a 96‐well Microplate Reader (Fisher Scientific Multiskan). The activity of ABCB1 (or ABCG2) ATPase specifically motivated by KSQ‐4279 was valued as the final deviations between the released inorganic phosphate with or without Na_3_VO_4_. The released inorganic phosphate could be directly observed from the standard curve.

### 4.8 Photoaffinity Labeling of ABCB1 or ABCG2 With [^125^I]‐IAAP

The assays were carried out in the light of our protocol established previously [27]. In brief, ABCB1‐expressing (or ABCG2‐expressing) crude membrane, derived from High Five insect cells (50 µg protein), was treated by KSQ‐4279 (0–20 µM) in Tris‐HCl (pH 7.5, 50 mM) for 5 min. Note that 3 nM [^125^I]‐IAAP (2200 Ci/nmol) was then put into the above reaction system and mixed for another 5‐min incubation away from hard light. The transporter proteins were radiolabeled by UV‐cross‐linking (on the ice, at 365 nm), followed by immunoprecipitating the radiolabeled ABCB1 (or ABCG2) by using antibody for C219 (Novus, Centennial, CO, USA) or BXP21 (Santa Cruz, Dallas, TX, USA). Afterward, samples were separated in a Tris‐acetate NuPAGE gel (7%), exposure to Eastman Kodak Bio‐Max MR film for drying overnight at −80°C. Finally, the incorporated radioactivity of ABCB1 (or ABCG2) was assessed via instrument of Storm 860 PhosphorImager system.

### 4.9 Western Blot Analysis

Drug treated cells were gathered and lysed in RIPA for extracting the cellular total proteins. Protein concentration of the lysates was tested by using Kit of Pierce BCA regents. Then equal amounts of total protein from different groups were submitted to SDS‐PAGE electrophoresing isolation, and subjected to transferring by using PVDF membranes (0.2 µm), followed by a 1–2 h blocking in skimmed milk (5%). The processed membranes were then probed by specific primary antibodies recognizing the proteins of interest at 4°C overnight. The specific protein bands were visualized by the chemiluminescent ECL Substrate (1705061, BIO‐RAD) after incubating with the corresponding secondary antibodies.

### 4.10 qRT‐PCR

First, total RNA was prepared by using the RNA Purification Kit (B0004D, EZBioscience, Roseville, MN, USA). Second, the RNA was reversed to the cDNA using a Mix (EZB‐RT2GQ, EZBioscience). Then, cDNA was subjected to the following assays of qRT‐PCR using SYBR Master Mix (A0001‐R2, EZBioscience). Real‐time PCR reactions were conducted as per the manufacturer's instructions. Sequences of the used primers are listed in Table S3.

### 4.11 Detecting the Amounts of ABCB1, ABCG2, or ABCC1 on Cell Membrane

Cancer cells, treated by 10 µM KSQ‐4279 or DMSO for 48 h, were gathered and washed by PBS. Then, cells were resuspended with PBS (50 µL, containing 1% FBS), and 10 µL FITC‐tagged ABCB1 antibody (11‐2439‐42, ThermoFisher), FITC‐tagged ABCG2 antibody (332014, Biolegend), or PE‐tagged ABCC1 antibody (IC19291P, R&D) was added for a 45‐min labeling at 4°C. Afterward, the cell sediment was re‐washed and resuspended in PBS (300 µL) for flow cytometric measurement of cell surface expression of the desired transporter.

### 4.12 Visualization of ABCB1, ABCG2, or ABCC1 Localized in Cells by Immunofluorescence

Following 48‐h incubation with or without 10 µM KSQ‐4279, cancer cells were washed by cold PBS, and incubated with paraformaldehyde (PFA, 4%) for 20 min to keep cells in the natural and organized state, then subjected to a 10‐min permeabilization by Triton X‐100 (0.1%). Afterward, the PBS‐washed cells were blocked in the bovine serum albumin (BSA) solution (3%, 30 min) and immunolabeled at 4°C overnight with 1% ABCB1 antibody (22336‐1‐AP, Proteintech), 1% ABCG2 antibody (ab229193, Abcam), or 2% ABCC1 antibody (sc‐18835, Santa Cruz). Finally, the signaling of primary antibodies was amplified by the 1‐h incubation with Alexa Fluor Plus 594‐conjugated IgG (1:1000) in the dark. Additionally, cell nuclei were further labeled by DAPI (1:1000) for 10–20 min. Finally, fluorescent images of cells were captured by using a Zeiss LSM 880 microscope.

### 4.13 Molecular Docking

As a widely employed approach, computer molecular docking efficiently predicts the binding action between compounds and proteins. In our work, KSQ‐4279, paclitaxel, or vincristine was docked to the substrate‐binding region of ABCB1 as follows: obtaining the 3D structure of molecular (KSQ‐4279, paclitaxel, and vincristine) from web of PubChem, and obtaining the crystal structure of ABCB1 protein (PDB: 6c0v) from the Protein Data Bank, then performing docking by software of AutoDock Vina [56]. Besides, the docking of KSQ‐4279, mitoxantrone, or topotecan to ABCG2 protein (PDB: 6ffc) and docking of KSQ‐4279 or doxorubicin to ABCC1 protein (PDB: 6uy0) were carried out similarly.

### 4.14 CETSA

CETSA is commonly used to verify the direct binding of drugs with target proteins based on the principle that ligand's binding increases the thermal stabilization of target proteins [57]. To confirm whether KSQ‐4279 binds to ABCB1, ABCG2, and ABCC1 proteins, CETSAs were carried out in KBv200, S1‐MI‐80, and HL60/adr cell lysates. Briefly, 2 × 10^7^ cells were collected and resuspended in PBS supplemented with protease inhibitor, then the suspensions were freeze‐thawed five times in liquid nitrogen, followed by a 20‐min centrifugation at 15,000 *g* and 4°C to separate the cell lysates. The lysates were divided into two aliquots and incubated with KSQ‐4279 (10 µM) or equal volumes of DMSO for 30 min at room temperature; then, lysates were divided into five aliquots and heated individually at the designated temperatures for 3 min. Finally, all heated lysates were centrifuged at 20,000 *g* for 20 min at 4°C to separate the soluble ligand‐bound proteins from the precipitates, followed by further analysis via western blot.

### 4.15 Statistical Analysis

Results in this work were confirmed by at least three independent repetitions of experiments. Data for experiments are defined as the mean ± standard deviation (SD). The statistical differences between two data sets were figured out by the method of Student's *t*‐test, and the difference was considered statistically significant at *p* < 0.05 (*p < 0.05; **p < 0.01; ***p < 0.001).

## Author Contributions

**Qihong Yang**: investigation, validation, writing – original draft, review, and editing. **Kewang Luo**: investigation, validation, writing – original draft, review, and editing. **Kenneth Kin Wah To**: investigation, validation, writing – original draft, review, and editing. **Can Pan**: formal analysis, software, visualization. **Sijia Li**: formal analysis, software, visualization. **Shuangli Zhu**: formal analysis, software, visualization. **Kai Fu**: formal analysis, software, visualization. **Fang Wang**: methodology, data curation, project administration. **Chuanan Wu**: conceptualization, resources, supervision. **Liwu Fu**: conceptualization, resources, supervision. The final manuscript has been read and approved by all authors.

## Funding

This work was funded by the National Key R&D Program of China (2022YFE0209700) and the National Natural Science Foundation of China (U21A20421, 82073882).

## Ethics Statement

This work strictly followed the ethical principles of Declaration of Helsinki. The animal experiments were approved by the Animal Ethics Committee of Sun Yat‐sen University Cancer Center (approval number: L102042023100C). The ethical approval for collection and use of patient‐derived tumor specimens was also granted by Sun Yat‐sen University Cancer Center (approval number: B2024‐406‐01).

## Conflicts of Interest

The authors declare no conflicts of interest.

## Supporting information



Table S1 KSQ‐4279 reversed MDR in ABCB1/ABCG2/ABCC1‐overexpressing cancer cells.
Table S2 KSQ‐4279 reversed MDR in ABCB1/ABCG2/ABCC1 stable‐transfected HEK293 cells.
Table S3 The sequences of the primers used for qRT‐PCR.
Figure S1. Molecular docking 2D drawings to show the interaction of ABCB1 protein with KSQ‐4279 (A), paclitaxel (B), vincristine (C); the interaction of ABCG2 protein with KSQ‐4279 (D), mitoxantrone (E), topotecan (F); and the interaction of ABCC1 protein with KSQ‐4279 (G), doxorubicin (H).

## Data Availability

Upon reasonable request, all data presented here are accessible from the corresponding authors.
