# Supplementary material for: KSQ‐4279, an Inhibitor of Ubiquitin Specific Peptidase 1, Enhanced the Chemotherapeutic Efficacy in ABCB1/ABCG2/ABCC1‐Mediated Multidrug Resistant Cancers
Source: MedComm (2020). 2025 Nov 29;6(12):e70517. doi: 10.1002/mco2.70517 (PMC12664909; doi:10.1002/mco2.70517)
Supplement: Supplementary file 1 — Table S1 KSQ‐4279 reversed MDR in ABCB1/ABCG2/ABCC1‐overexpressing cancer cells. Table S2 KSQ‐4279 reversed MDR in ABCB1/ABCG2/ABCC1 stable‐transfected HEK293 cells. Table S3 The sequences of the primers used for qRT‐PCR. Figure S1. Molecular docking 2D drawings to show the interaction of ABCB1 protein with KSQ‐4279 (A), paclitaxel (B), vincristine (C); the interaction of ABCG2 protein with KSQ‐4279 (D), mitoxantrone (E), topotecan (F); and the interaction of ABCC1 protein with KSQ‐4279 (G), doxorubicin (H). [file MCO2-6-e70517-s001.pdf]

KSQ-4279, an inhibitor of ubiquitin specific peptidase 1, enhanced the chemotherapeutic efficacy in ABCB1/ABCG2/ABCC1-mediated multidrug resistant cancers

Supplementary Information

Qihong Yang [1, 2, #], Kewang Luo [2, #], Kenneth Kin Wah To [3, #], Can Pan [1], Kai Fu [1], Shuangli Zhu [1], Sijia Li [1], Fang Wang [1], Chuanan Wu [2, *], Liwu Fu [1, *]

[1] *State Key Laboratory of Oncology in South China, Guangdong Provincial Clinical Research Center for Cancer, Sun Yat-sen University Cancer Center, Guangzhou 510060, P. R. China.*

[2] *People's Hospital of Longhua, Shenzhen 518109, China.*

[3] *School of Pharmacy, The Chinese University of Hong Kong, Hong Kong 999077, P.R. China.*

* Correspondence: Chuanan Wu (wuca@163.com); Liwu Fu (fulw@mail.sysu.edu.cn).

[#] These authors contributed equally to this work.

Supplementary Tables

Table S1 KSQ-4279 reversed MDR in ABCB1/ABCG2/ABCC1-overexpressing cancer cells

Compounds	IC_{50} ± SD (μM) (fold-reversal)			
	**KB**		**KBv200 (ABCB1)**	
Paclitaxel	0.00672 ± 0.00073	(1.00)	0.13646 ± 0.01081	(1.00)
+ KSQ-4279 (2.5 μM)	0.00547 ± 0.00039	(1.23)	0.02149 ± 0.00712	(6.35) ***
+ KSQ-4279 (5 μM)	0.00520 ± 0.00024	(1.29)	0.01368 ± 0.00014	(9.97) ***
+ KSQ-4279 (10 μM)	0.00584 ± 0.00006	(1.15)	0.00595 ± 0.00114	(22.94) ***
+ Verapamil (10 μM)	0.00557 ± 0.00058	(1.21)	0.00740 ± 0.00067	(18.43) ***
Vincristine	0.00203 ± 0.00012	(1.00)	0.12651 ± 0.03227	(1.00)
+ KSQ-4279 (2.5 μM)	0.00187 ± 0.00006	(1.09)	0.00932 ± 0.00102	(13.58) ***
+ KSQ-4279 (5 μM)	0.00178 ± 0.00005	(1.14)	0.00336 ± 0.00118	(37.62) ***
+ KSQ-4279 (10 μM)	0.00177 ± 0.00007	(1.15)	0.00173 ± 0.00013	(73.29) ***
+ Verapamil (10 μM)	0.00174 ± 0.00004	(1.17)	0.00088 ± 0.00012	(143.33) ***
Doxorubicin	0.02454 ± 0.00246	(1.00)	7.78204 ± 0.29419	(1.00)
+ KSQ-4279 (2.5 μM)	0.02152 ± 0.00175	(1.14)	0.59779 ± 0.07723	(13.02) ***
+ KSQ-4279 (5 μM)	0.01863 ± 0.00102	(1.32)	0.40501 ± 0.07206	(19.21) ***
+ KSQ-4279 (10 μM)	0.01813 ± 0.00105	(1.35)	0.28650 ± 0.01665	(27.16) ***
+ Verapamil (10 μM)	0.02358 ± 0.00186	(1.04)	0.38590 ± 0.06489	(20.17) ***
Cisplatin	1.21335 ± 0.11206	(1.00)	1.47599 ± 0.26253	(1.00)
+ KSQ-4279 (10 μM)	1.05428 ± 0.09016	(1.15)	1.42317 ± 0.10906	(1.04)
	**MCF-7**		**MCF-7/adr (ABCB1)**	
Doxorubicin	0.58360 ± 0.04391	(1.00)	6.85493 ± 2.41642	(1.00)
+ KSQ-4279 (2.5 μM)	0.65772 ± 0.07363	(0.89)	4.66000 ± 0.64353	(1.47) *
+ KSQ-4279 (5 μM)	0.59770 ± 0.17991	(0.98)	3.32114 ± 0.54169	(2.06) **
+ KSQ-4279 (10 μM)	0.60959 ± 0.13979	(0.96)	0.94729 ± 0.14565	(7.24) ***
+ Verapamil (10 μM)	0.57540 ± 0.11703	(1.01)	1.20966 ± 0.09561	(5.67) ***
Cisplatin	20.05109 ± 0.83214	(1.00)	26.45063 ± 1.70869	(1.00)
+ KSQ-4279 (10 μM)	24.68168 ± 0.98383	(0.81)	29.78348 ± 1.78452	(0.89)
	**S1**		**S1-MI-80 (ABCG2)**	
Mitoxantrone	0.09724 ± 0.00877	(1.00)	9.71894 ± 2.10491	(1.00)
+ KSQ-4279 (2.5 μM)	0.07723 ± 0.00835	(1.26)	9.40638 ± 1.70973	(1.03)
+ KSQ-4279 (5 μM)	0.08901 ± 0.00090	(1.09)	6.74545 ± 2.58673	(1.44) *
+ KSQ-4279 (10 μM)	0.08741 ± 0.01012	(1.11)	2.43530 ± 0.25795	(3.99) ***
+ Ko143 (50 nM)	0.10222 ± 0.01325	(0.95)	2.87135 ± 0.84374	(3.38) ***
Topotecan	1.54006 ± 0.14151	(1.00)	36.20099 ± 5.09340	(1.00)
+ KSQ-4279 (2.5 μM)	1.29579 ± 0.20043	(1.19)	36.38538 ± 2.82176	(0.99)
+ KSQ-4279 (5 μM)	1.46514 ± 0.37248	(1.05)	9.28717 ± 3.16821	(3.90) ***
+ KSQ-4279 (10 μM)	1.49283 ± 0.18297	(1.03)	3.67751 ± 0.56127	(9.84) ***
+ Ko143 (100 nM)	1.38275 ± 0.24371	(1.11)	17.94800 ± 2.01241	(2.02) **

Cisplatin	28.77122 ± 1.25119	(1.00)	30.28132 ± 1.82832	(1.00)
+ KSQ-4279 (10 μM)	30.40940 ± 1.45062	(0.95)	31.90803 ± 1.44046	(0.95)
	**H460**		**H460/MX20 (ABCG2)**	
Mitoxantrone	0.05348 ± 0.00274	(1.00)	1.26483 ± 0.13239	(1.00)
+ KSQ-4279 (2.5 μM)	0.04986 ± 0.00284	(1.07)	1.35760 ± 0.07187	(0.93)
+ KSQ-4279 (5 μM)	0.06417 ± 0.01472	(0.83)	0.63145 ± 0.11718	(2.00) **
+ KSQ-4279 (10 μM)	0.06243 ± 0.00267	(0.86)	0.42549 ± 0.04178	(2.97) ***
+ Ko143 (30 nM)	0.05323 ± 0.00713	(1.00)	0.28734 ± 0.11836	(4.40) ***
Cisplatin	4.60214 ± 1.09861	(1.00)	12.76340 ± 2.67312	(1.00)
+ KSQ-4279 (10 μM)	4.19898 ± 0.60082	(1.10)	12.69953 ± 2.19950	(1.01)
	**HL60**		**HL60/adr (ABCC1)**	
Doxorubicin	0.01955 ± 0.00140	(1.00)	3.40322 ± 0.21419	(1.00)
+ KSQ-4279 (2.5 μM)	0.01850 ± 0.00274	(1.06)	2.39713 ± 0.19004	(1.42) *
+ KSQ-4279 (5 μM)	0.01921 ± 0.00115	(1.02)	1.44056 ± 0.39354	(2.36) ***
+ KSQ-4279 (10 μM)	0.02096 ± 0.00342	(0.93)	0.93975 ± 0.14153	(3.62) ***
+ MK571 (20 μM)	0.01978 ± 0.00297	(0.99)	1.37911 ± 0.20588	(2.47) ***
Cisplatin	0.59427 ± 0.12335	(1.00)	1.40261 ± 0.17131	(1.00)
+ KSQ-4279 (10 μM)	0.58839 ± 0.12807	(1.01)	1.42633 ± 0.25115	(0.98)
	**SW1573**		**SW1573/2R120 (LRP)**	
Doxorubicin	0.04042 ± 0.00085	(1.00)	0.59309 ± 0.01696	(1.00)
+ KSQ-4279 (2.5 μM)	0.03517 ± 0.00885	(1.15)	0.71794 ± 0.21736	(0.83)
+ KSQ-4279 (5 μM)	0.04334 ± 0.00772	(0.93)	0.69672 ± 0.12201	(0.85)
+ KSQ-4279 (10 μM)	0.04671 ± 0.00250	(0.87)	0.59031 ± 0.21890	(1.00)
	**HEK293/Vector**		**HEK293/MRP7-2**	
Paclitaxel	0.06823 ± 0.01204	(1.00)	1.24046 ± 0.26714	(1.00)
+ KSQ-4279 (2.5 μM)	0.06567 ± 0.01071	(1.04)	1.09420 ± 0.24421	(1.13)
+ KSQ-4279 (5 μM)	0.06415 ± 0.01123	(1.06)	1.10154 ± 0.25335	(1.13)
+ KSQ-4279 (10 μM)	0.07171 ± 0.00844	(0.95)	1.13424 ± 0.29758	(1.09)

Cell survival was evaluated by MTT assay. Data was reported as Means ± SD. (*, $P < 0.05$; **, $P < 0.01$; ***, $P < 0.001$. Compared with the corresponding values obtained in the absence of inhibitor). The MDR reversal activity of KSQ-4279 in MDR cells was calculated by dividing the IC_{50} of cells treated only with anticancer drugs by that co-treated with drugs and KSQ-4279. Verapamil (a definite ABCB1 inhibitor), Ko143 (a definite ABCG2 inhibitor) or MK571 (a definite ABCC1 inhibitor) were used as the positive control, and cisplatin was as a non-substrate control.

Table S2 KSQ-4279 reversed MDR in ABCB1/ABCG2/ABCC1 stable-transfected HEK293 cells

Compounds	IC50 ± SD (μM) (fold-reversal)			
	**HEK293/Vector**		**HEK293/ABCB1**	
Paclitaxel	0.06823 ± 0.01204	(1.00)	1.11502 ± 0.30638	(1.00)
+ KSQ-4279 (2.5 μM)	0.06567 ± 0.01071	(1.04)	0.55117 ± 0.12892	(2.02) **
+ KSQ-4279 (5 μM)	0.06415 ± 0.01123	(1.06)	0.38734 ± 0.09750	(2.88) **
+ KSQ-4279 (10 μM)	0.07171 ± 0.00844	(0.95)	0.17582 ± 0.03515	(6.34) ***
+ Verapamil (10 μM)	0.06070 ± 0.00555	(1.12)	0.07446 ± 0.01447	(14.98) ***
Doxorubicin	0.11912 ± 0.02515	(1.00)	1.27208 ± 0.17045	(1.00)
+ KSQ-4279 (2.5 μM)	0.11677 ± 0.02473	(1.02)	1.05744 ± 0.17630	(1.20)
+ KSQ-4279 (5 μM)	0.11702 ± 0.01212	(1.02)	0.60826 ± 0.01779	(2.09) **
+ KSQ-4279 (10 μM)	0.12506 ± 0.01565	(0.95)	0.16083 ± 0.04838	(7.91) ***
+ Verapamil (10 μM)	0.13289 ± 0.02545	(0.90)	0.12869 ± 0.03399	(9.88) ***
Cisplatin	16.17682 ± 1.85487	(1.00)	26.10736 ± 3.11130	(1.00)
+ KSQ-4279 (10 μM)	18.60621 ± 4.94647	(0.87)	28.67973 ± 1.25449	(0.91)
	**HEK293/Vector**		**HEK293/ABCG2**	
Mitoxantrone	0.24218 ± 0.01964	(1.00)	3.21068 ± 0.20074	(1.00)
+ KSQ-4279 (2.5 μM)	0.27872 ± 0.03354	(0.87)	3.44221 ± 0.89232	(0.93)
+ KSQ-4279 (5 μM)	0.23914 ± 0.00966	(1.01)	1.65159 ± 0.39163	(1.94) **
+ KSQ-4279 (10 μM)	0.22461 ± 0.05577	(1.08)	0.87898 ± 0.22726	(3.65) ***
+ Ko143 (100 nM)	0.20760 ± 0.06184	(1.17)	0.67910 ± 0.09112	(4.73) ***
Cisplatin	17.32465 ± 2.61898	(1.00)	21.08623 ± 1.77676	(1.00)
+ KSQ-4279 (10 μM)	19.07483 ± 0.49734	(0.91)	19.10386 ± 2.81107	(1.10)
	**HEK293/Vector**		**HEK293/ABCC1**	
Doxorubicin	0.01446 ± 0.00473	(1.00)	0.23705 ± 0.03562	(1.00)
+ KSQ-4279 (2.5 μM)	0.01482 ± 0.00269	(0.98)	0.16802 ± 0.03287	(1.41) *
+ KSQ-4279 (5 μM)	0.01647 ± 0.00532	(0.88)	0.07922 ± 0.02337	(2.99) ***
+ KSQ-4279 (10 μM)	0.01519 ± 0.00377	(0.95)	0.04839 ± 0.00672	(4.90) ***
+ MK571 (20 μM)	0.01315 ± 0.00257	(1.10)	0.04250 ± 0.01599	(5.58) ***
Cisplatin	2.15723 ± 0.32833	(1.00)	4.16338 ± 0.50499	(1.00)
+ KSQ-4279 (10 μM)	2.37428 ± 0.18410	(0.91)	4.21689 ± 0.25784	(0.99)

Cell survival was evaluated by MTT assay. Data was reported as Means ± SD. (*, $P < 0.05$; **, $P < 0.01$; ***, $P < 0.001$. Compared with the corresponding values obtained in the absence of inhibitor). The MDR reversal activity of KSQ-4279 in MDR cells was calculated by dividing the IC_{50} of cells treated only with anticancer drugs by that co-treated with drugs and KSQ-4279. Verapamil (a definite ABCB1 inhibitor), Ko143 (a definite ABCG2 inhibitor) or MK571 (a definite ABCC1 inhibitor) were used as the positive control; and cisplatin was as a non-substrate control.

Table S3 The sequences of the primers used for qRT-PCR

Gene	Sense (5′-3′)	Antisense (5′-3′)
GAPDH	GTCTCCTCTGACTTCAACAGCG	ACCACCCTGTTGCTGTAGCCAA
ABCB1	CAGGCTTGCTGTAATTACCCA	TCAAAGAAACAACGGTTCGG
ABCG2	TGGCTGTCATGGCTTCAGTA	GCCACGTGATTCTTCCACAA
ABCC1	ATGTCACGTGGAATACCAGC	GAAGACTGAACTCCCTTCCT

Supplementary Figures

Figure S1

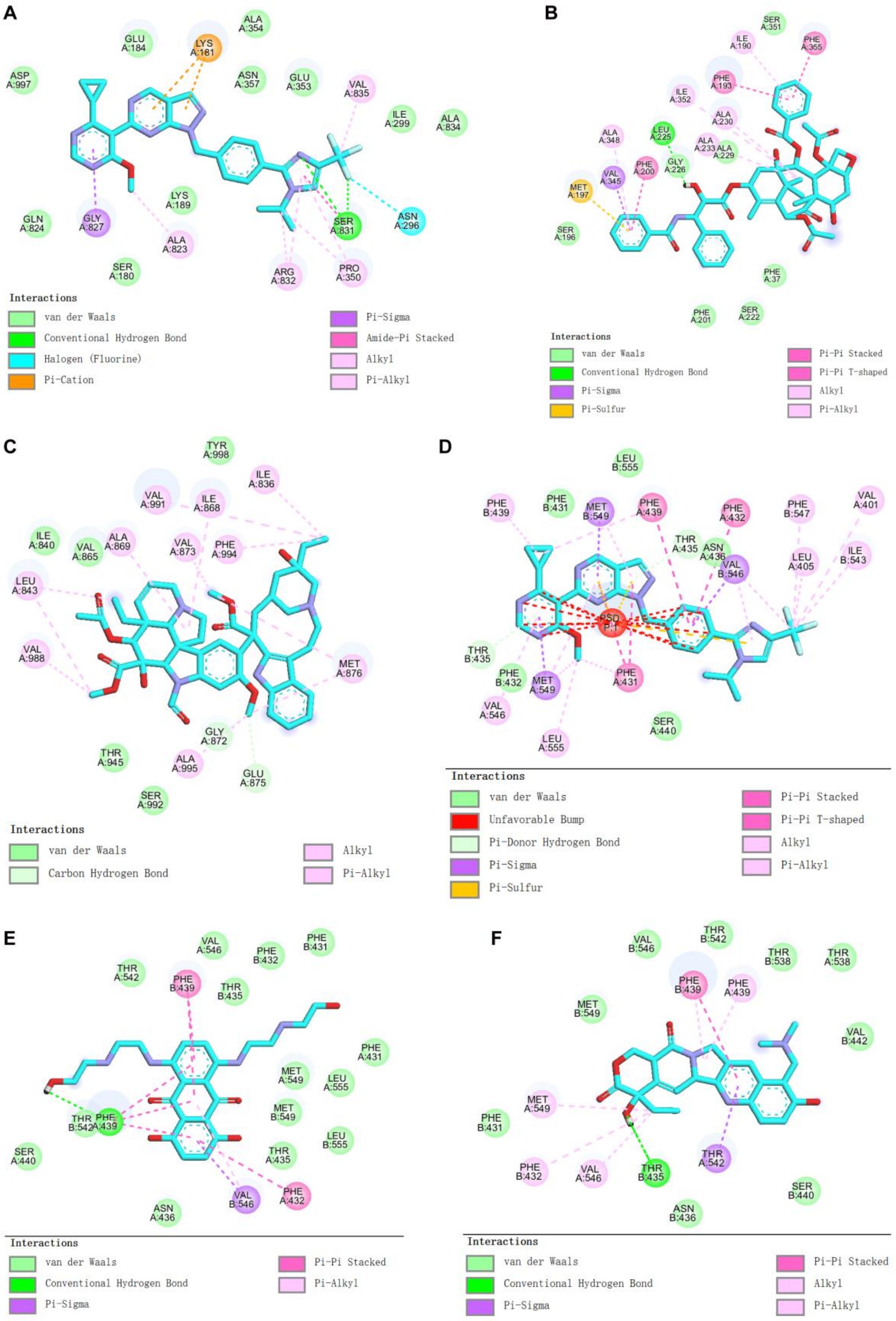

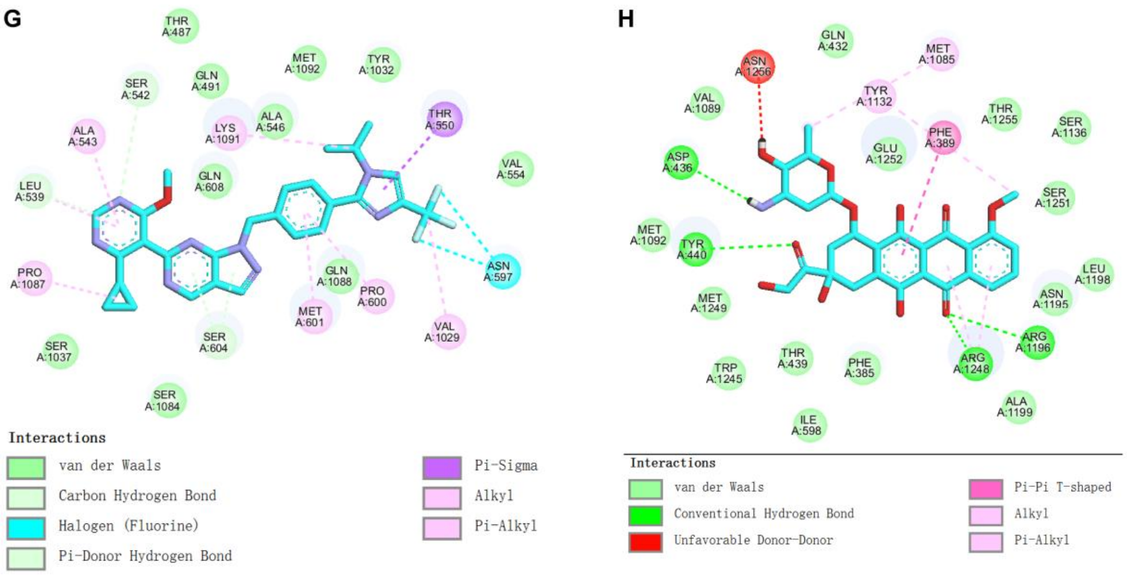

Figure S1. Molecular docking 2D drawings to show the interaction of ABCB1 protein with KSQ-4279 **(A)**, paclitaxel **(B)**, vincristine **(C)**; the interaction of ABCG2 protein with KSQ-4279 **(D)**, mitoxantrone **(E)**, topotecan **(F)**; and the interaction of ABCC1 protein with KSQ-4279 **(G)**, doxorubicin **(H)**.
